# Manganese: biology, physiology and role in disease

**DOI:** 10.1038/s41421-026-00894-5

**Published:** 2026-06-16

**Authors:** Zhidan Xia, Xinran Li, Rui Liu, Karin Tuschl, Junxia Min, Fudi Wang

**Affiliations:** 1 The Second Affiliated Hospital, School of Public Health, State Key Laboratory of Experimental Hematology, Zhejiang University School of Medicine, Hangzhou, Zhejiang China; 2 The First Affiliated Hospital, Institute of Translational Medicine, Zhejiang University School of Medicine, Hangzhou, Zhejiang China; 3 UCL Great Ormond Street Institute of Child Health, University College London, London, UK

**Keywords:** Mechanisms of disease, Ion channel signalling

## Abstract

Manganese (Mn) has lingered in the shadows as a mere enzymatic cofactor, with its profound role in regulating the most fundamental life processes largely overlooked. This review heralds a “manganese renaissance” — a paradigm shift that elevates Mn from a passive trace element to a dynamic architect of metabolic homeostasis and a critical driver of disease. We synthesize breakthroughs that redefine its biological significance. In addition to enabling reactions for enzymes such as MnSOD, Mn actively governs lipid trafficking via the modulation of the COPII complex, facilitates cGAS/STING signaling for host immune responses, and precisely activates ion transporters and sensors to maintain cellular homeostasis. Dysregulated Mn homeostasis — whether stemming from genetic defects in key transporters (SLC30A10, SLC39A8, SLC39A11, and SLC39A14) or environmentally induced overload — fuels a spectrum of pathologies, including metabolic syndrome, Parkinsonism-like neurodegeneration, hepatic dysfunction, cardiovascular disease, and immune dysfunction. This disruption underscores the irreplaceable role of Mn as a biological linchpin, as its balance is not merely supportive but also central to sustaining health. In the future, we outline translational frontiers — from dietary Mn modulation and transporter-specific therapies for genetic Mn disorders to the elucidation of Mn signaling and the development of exposure guidelines to safeguard public health. This synthesis reaffirms that Mn is far more important than simply functioning as a nutrient. Research into Mn functions has been conducted across biology, environmental science, and medicine, and Mn acts as a master regulator whose emerging mechanisms will reshape our understanding of metabolic health and disease pathogenesis.

## Introduction

Manganese (Mn), one of the most abundant metals on Earth^1^, is an essential trace element in all living organisms and enables key enzymatic functions and cellular signaling^2,3^. However, excess Mn exposure has significant health risks^4^. Thus, higher organisms use coordinated organ systems and specialized proteins to regulate the uptake, distribution, and excretion of Mn to maintain systemic homeostasis and balance these dual roles in health and disease^5^. Over the past decade, advances in the study of the biological roles of Mn have reshaped our understanding of its role in health and disease, with ongoing research continuously refining our understanding of its metabolic pathways and therapeutic implications^6,7^.

**Fig. 1:**
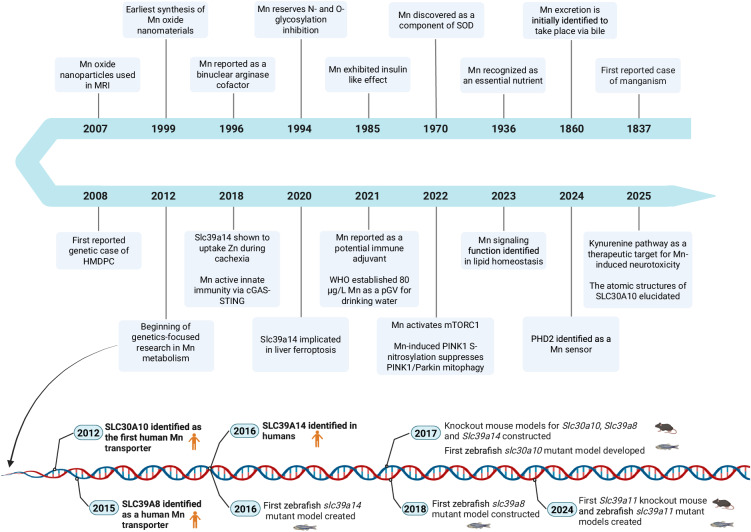
Milestones in manganese research.

Previous reviews have discussed Mn physiology alongside Mn-associated neurotoxicity, focusing on its effects on health and disease^8,9^; these reviews have noted that Mn-dependent mitochondrial redox control is a possible mechanistic link between Mn status and disease vulnerability^10^ and have summarized how chemical tools for tracking Mn have helped map its subcellular localization, speciation, and dynamics^11^. Here, we provide a comprehensive overview of the complex interplay between Mn metabolism and various diseases, integrating insights gleaned from environmental Mn toxicity, inherited Mn transportopathies, and their molecular mechanisms. We also summarize the knowledge regarding the Mn distribution in various human tissues, analyze the physiological roles of Mn transporters in mammalian systems, and delineate the consequences of their dysfunction with respect to disease. In addition, we highlight various Mn-mediated molecular pathways in terms of their physiological context and provide a contrast to the toxicological effects and molecular regulatory responses to excessive exposure to environmental Mn. We then summarize reported cases of patients with biallelic pathogenic variants in Mn transporter genes, detailing their phenotypes and therapeutic interventions, as well as relatively common diseases linked to an Mn imbalance. Finally, we identify critical questions and propose future research designed to provide important new insights into Mn biology and pathophysiology. Indeed, the rapidly evolving field of Mn metabolism and its implications in disease continue to provide valuable new information (Fig. 1 and Box 1).

Box 1 Milestones in manganese research **Basics of manganese physiology**

In 1860, Von Oethingen discovered that bile is the primary pathway for excreting manganese (Mn)^28^, and in 1935, Mn was shown to be essential for mammalian physiology^315^. However, a truly pivotal breakthrough was reported in 1970 when Fridovich’s group purified Mn-containing superoxide dismutase (SOD) in *E. coli*, establishing Mn as a critical enzyme cofactor^316^. Subsequently, Christianson’s group revealed a unique binuclear Mn²⁺–Mn²⁺ active-site cluster that underpins Mn-dependent catalysis by arginase^317^. In 1985, Subasinghe et al. first found that Mn exerts an insulin-mimetic effect on hepatic glucose metabolism^318^. In 1994, Kaufman et al. showed that Mn plays a role in *N*-linked and *O*-linked glycosylation in mammalian cells^319^. Recently, Chen and colleagues reported that Mn plays a role in coat protein complex II (COPII) condensation and lipid homeostasis^136^.

**Health risks and regulatory functions**

Mn research has evolved significantly since the first documented report of Mn-induced toxicity (“manganism”) by the Scottish physician John Couper in 1837^1^. Prolonged or excessive Mn exposure causes neurotoxicity, in which Mn-induced dysregulation of mitophagy and autophagy is a contributing mechanism^144^. In 2022, Xu and colleagues showed that Mn induces the *S*-nitrosylation of PINK1, leading to neuronal damage by repressing PINK1/Parkin-mediated mitophagy^320^, and Wellinger and colleagues showed that Mn directly shapes autophagy by activating target of rapamycin complex 1 (TORC1, mTORC1 in mammals), likely by lowering the *K*_m_ of TORC1 for ATP to improve ATP coordination in the catalytic cleft^321^. The World Health Organization established a provisional guideline value of 80 µg/L of Mn in drinking water to avoid poisoning in 2021^322^. Recently, Mukhopadhyay and colleagues identified the enzyme prolyl hydroxylase domain 2 (PHD2) as a cellular Mn sensor^139^ and found that pharmacologically inhibiting kynurenine metabolism reduced Mn-induced neuromotor deficits^142^.

**Nanotechnology and medical applications**

Advances in nanotechnology also expanded the use of Mn in clinical applications. For example, in 1999, Alivisatos and colleagues synthesized monodisperse Mn oxide nanomaterials^323^, while Hyeon and colleagues pioneered the development of Mn oxide nanoparticles in 2007 for use in in vivo magnetic resonance imaging (MRI)^324^. In 2018, Jiang and colleagues found that Mn activates anti-viral innate immunity via the cGAS–STING pathway^2^, and in 2021, they developed a colloidal Mn salt (MnJ) as a potent immune adjuvant^280^.

**Genetic and metabolic insights**

In 2008, Clayton and colleagues identified the first genetic basis of hypermanganesemia with dystonia, polycythemia, and cirrhosis (HMDPC) syndrome^199^, thereby catalyzing the study of genetics in the context of Mn metabolism. This finding led to the subsequent discovery of key Mn transporters. First, the transporter SLC30A10 (also known as ZnT10) was identified independently by the Bonifati and Tuschl groups in 2012^84,85^, then subsequently shown by the Mukhopadhyay group and our group to play a critical role in systemic Mn homeostasis in mouse and zebrafish models, respectively^35,36^. In 2025, the Yang lab resolved high-resolution cryo-EM structures of SLC30A10 and identified a previously unrecognized Mn²⁺-specific binding site (D40/N127/D248/S252)^93^. Second, the transporter SLC39A8 (also known as ZIP8) was linked to Mn regulation in 2015 by the Abou Jamra and Marquardt groups^209,214^; subsequently, Gurnett and colleagues used a zebrafish model to link this transporter to scoliosis^46^, while Rader and colleagues used mouse models to link SLC39A8 to Mn-dependent enzyme modulation^16^. The third transporter, SLC39A14 (ZIP14), was first shown in 2016 by Tuschl et al.^52^ to play a role in Mn homeostasis; the subsequent use of various mouse models by the Cousins, Mukhopadhyay, Wang, and Knutson groups confirmed its role in systemic Mn homeostasis^48–51^, while the Acharyya group and our group showed links between this transporter and zinc uptake^325^ and liver ferroptosis^220^, respectively. Finally, we recently identified SLC39A11 (ZIP11) as the fourth Mn transporter using zebrafish and mouse models, thus warranting future studies regarding the putative link between this transporter and disease^54^.

This long timeline spanning nearly two centuries underscores the dual roles of Mn as both an essential nutrient and a potential toxin, driving interdisciplinary studies designed to unravel its complex physiology and its therapeutic potential (summarized in Fig. 1).

## Systemic manganese homeostasis

### The physiological distribution of Mn

**Fig. 2:**
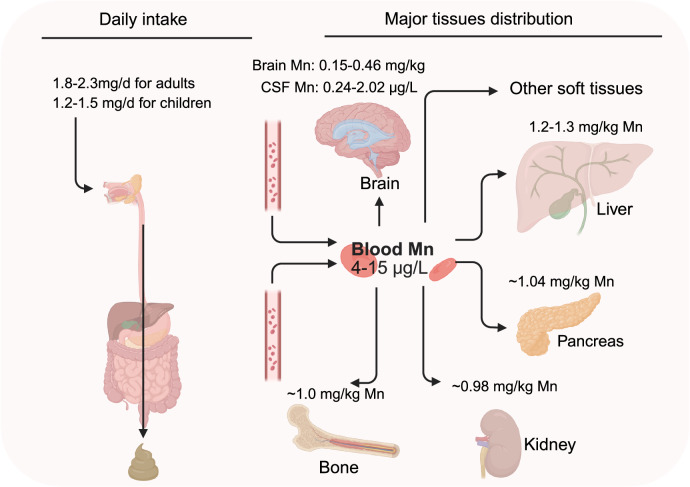
Systemic manganese distribution and homeostasis in humans. The daily levels of Mn intake may be 1.8–2.3 mg for adults and 1.2–1.5 mg for young children. Under physiological conditions, absorbed Mn enters the circulation and is distributed to various target tissues and organs, including the brain, hepatobiliary system, pancreas, kidneys, and bone. Mn homeostasis is maintained through the biliary excretion of excess Mn, which is subsequently eliminated in the feces.

Mn plays essential physiological roles in maintaining human health^12^. The dietary sources of Mn are mainly plant-based foods, such as whole grains, nuts, seeds, legumes, most vegetables, and tea, while refined grains and animal-based foods contain relatively little Mn^13^. The gastrointestinal tract serves as the primary site for Mn absorption^14^. Upon its absorption, Mn rapidly enters the circulation and is distributed to various tissues to support their physiological functions, but the recommended daily intake of Mn has not been established, although reasonable intake levels may be 1.8–2.3 mg for adults and 1.2–1.5 mg for young children^13^. Notably, insufficient Mn intake has been associated with numerous pathological conditions, including impaired growth, skeletal abnormalities, glucose intolerance, and altered regulation of lipid/carbohydrate metabolism^15^. In patients with genetic disorders that affect Mn homeostasis, insufficient Mn levels can impair enzyme activity by limiting the availability of this essential catalytic cofactor, leading to impaired biological processes, immune dysfunction, and developmental delays^16^. However, given the abundance of Mn in drinking water, soil, and many foods, Mn deficiency is relatively rare in humans^17^. On the other hand, Mn-related pathologies that arise from Mn toxicity and metabolic disorders due to excessive Mn accumulation are relatively common. Under normal conditions, the tissue concentrations of Mn in humans are 4–15 mg/L, 0.15–0.46 mg/kg, 1.2–1.3 mg/kg, ~1.04 mg/kg, ~1 mg/kg, and ~0.98 mg/kg in the blood, brain, liver, pancreas, bone and kidneys, respectively^18^ (Fig. 2). Importantly, Mn levels are substantially lower than those of other trace elements, such as iron and zinc; for example, an adult typically contains 1.8 g and 1 g of iron in the blood and liver, respectively, and 2 mg and 100 mg of zinc in the blood and liver, respectively, compared with only 40 μg and 1.5 mg of Mn in the blood and liver, respectively^18–20^. Although present in relatively trace amounts, Mn performs unique and indispensable functions in innate immune activation and nanomedical applications, distinguishing it from other metal ions.

Under physiological conditions, approximately 40% of the total Mn stored in the body is accumulated in bone tissue, as determined in autopsy studies^21,22^. Mn plays critical roles in bone regeneration and mineralization and in maintaining skeletal strength^23^; however, several key questions remain regarding Mn dynamics in bone tissue, including whether bones serve as a Mn reservoir during nutritional deficiency and how the distribution of Mn in bone tissue affects systemic Mn homeostasis^24,25^.

Mn absorption occurs primarily in the small intestine, with relatively small amounts of Mn derived from the enterohepatic circulation and reabsorption via the renal tubules, thus maintaining systemic Mn balance^26,27^. Excessive Mn is eliminated predominantly through hepatobiliary fecal excretion^28,29^, with other excretion pathways (e.g., urine, sweat, and breast milk) playing a relatively minimal role. Notably, Mn is cleared from the cerebrospinal fluid particularly slowly^1,29–31^.

### Functions of manganese transporters

**Fig. 3:**
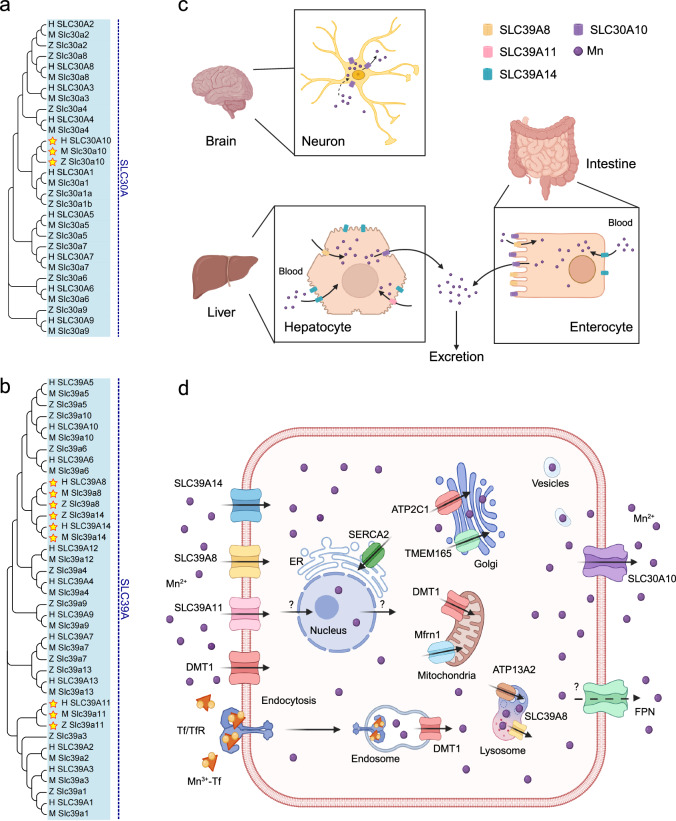
Mn homeostasis is tightly regulated by SLC metal transporters. **a**, **b** Phylogenetic analysis of the *SLC39A* and *SLC30A* gene families. The colored stars indicate known Mn transporters. H: human; M: mouse; Z: zebrafish. **c** Tissue-specific Mn transport mechanisms. SLC30A10 mediates Mn efflux in neurons, whereas SLC39A14 imports Mn into hepatocytes/enterocytes for SLC30A10-mediated biliary excretion. In contrast, SLC39A8 recaptures Mn from the bile and intestines. Finally, SLC39A11 may play a minor role in Mn uptake in the liver. **d** Cellular Mn transport pathways. SLC39A8, SLC39A14, and SLC39A11 mediate Mn^2+^ uptake, whereas the transferrin receptor (TfR) facilitates Mn^3+^ uptake via endocytosis. SLC30A10 exports Mn^2+^; ferroportin (FPN) may also play a role in Mn^2+^ efflux. With respect to the regulation of Mn in organelles, SERCA2, SPCA1, TMEM165, ATP13A2, Mfrn1, and DMT1 are involved in maintaining subcellular Mn homeostasis.

Manganese is both an essential trace element and a potential environmental heavy metal pollutant. Given these dual roles, precise regulatory systems are necessary to maintain Mn homeostasis in vivo, and Mn transporters act as the key executors of such regulation^32,33^. In contrast to our long-established knowledge regarding the effects of environmental Mn exposure, research regarding genetic disorders of Mn metabolism has emerged in just the past decade^34^. Since 2012, four genes encoding Mn transporters — *SLC30A10*, *SLC39A8*, *SLC39A11*, and *SLC39A14* — have been identified and characterized, and the existence of these evolutionarily conserved transporters indicates high functional conservation among species (Fig. 3a, b).

The discovery of Mn transporters led to studies of Mn metabolism at the systemic and genetic levels using genetic animal models. For example, Hutchens et al. reported that compared with control mice, *Slc30a10* knockout mice are much smaller and have significantly higher levels of systemic Mn, and unexpectedly, Mn accumulation caused severe hypothyroidism in these mice^35^, even though the thyroid is not a primary site of Mn accumulation in human patients. In contrast, *slc30a10*-deficient zebrafish fully recapitulate the symptoms observed in humans, including systemic Mn accumulation and subsequent neurological deficits, polycythemia, and liver injury^36^. Given that SLC30A10 is a putative Mn exporter and that mutations in *SLC30A10* lead to elevated systemic Mn levels in zebrafish, mice, and humans, an interesting question is which organ(s) are responsible for excreting Mn. Various conditional *Slc30a10* knockout mice have been generated to address this question. The high expression levels of *SLC30A10* in the brain, liver, and small intestine of adult humans suggest that this protein plays key functional roles in these tissues^37^; however, mice lacking *Slc30a10* specifically in neurons, the liver, or the small intestine exhibit minimal alterations in Mn levels^38,39^, although endoderm-specific *Slc30a10* knockout mice exhibit changes in systemic Mn levels that are similar to global *Slc30a10* knockout mice^38^. Together, these genetic mouse models suggest that additional endoderm-derived organs contribute to SLC30A10-mediated Mn metabolism. Interestingly, Ahmad et al. found that low levels of 12α-hydroxylated bile acids drive *Slc30a10* expression and cellular Mn efflux in mouse primary ileum organoids^40^, suggesting a close interaction between Mn and various metabolites in the intestine, thus warranting further study in the future.

Unlike the Mn-exporting function of SLC30A10, the LIV-1 subfamily transporters SLC39A8 and SLC39A14 are involved in Mn uptake, as determined using genetic animal models. First, hypomorphic *Slc39a8* mice exhibit impaired growth, multiple organ hypoplasia, anemia, and perinatal death, with decreased tissue levels of Zn and Fe; however, the authors did not measure Mn levels in this model^41^. Lin et al. reported that compared with control mice, inducible *Slc39a8* knockout (Slc39a8-iKO) and liver-specific *Slc39a8* knockout (Slc39a8-LSKO) mice had decreased Mn levels in the blood and in multiple organs but no changes in Zn or Fe levels^16^. The decreased Mn levels were more obvious in the Slc39a8-LSKO mice, and the overexpression of human *SLC39A8* in the mouse liver led to increased systemic Mn levels, indicating that hepatic SLC39A8 is a key regulator of systemic Mn homeostasis. Notably, the loss of function of Slc39a8 led to Mn deficiency, which directly reduced the activity of Mn-dependent enzymes, such as arginase, in the liver and kidney and disrupted serum protein *N*-glycosylation, which is linked to defective β-1,4-galactosyltransferase activity in mice. Importantly, the authors also showed that Slc39a8 localizes to the apical membrane of cholangiocytes and recovers Mn from bile, thereby reducing Mn excretion and maintaining systemic Mn storage^16^. Recently, Yu and Zhao reported that high dietary Mn intake leads to a decrease in Slc39a8 expression in the liver to prevent Mn overload in young mice but not in adult mice, suggesting an age-dependent difference in which hepatic *Slc39a8* regulates Mn metabolism^42^. More recently, Choi et al. observed that Slc39a8 is localized to the apical membrane and mediates ^54^Mn uptake in intestinal organoid monolayer cultures^43^. The authors also found that intestinal epithelial cell-specific *Slc39a8* knockout (Slc39a8-IEC KO) mice have markedly decreased Mn levels in the blood and in most organs and develop colitis; moreover, treating Slc39a8-IEC KO mice with an alkaline ceramidase 1 (ACER1, a key enzyme in sphingolipid metabolism) inhibitor attenuated colitis by restoring barrier function^43^. Together, these studies highlight the essential roles that SLC39A8 plays in intestinal Mn absorption and epithelial integrity and suggest a possible therapeutic target for inflammatory bowel disease (IBD) associated with impaired Mn homeostasis.

Given that the *SLC39A8* p.Ala391Thr (rs13107325) variant has been associated with more than 22 clinical conditions, an important question is how this single missense variant can cause such a wide range of pathologies^44^. For example, Nakata et al. showed that the altered function of Mn-dependent glycosyltransferase led to colon barrier dysfunction and inert inflammation in an SLC39A8^A391T^ mouse model, suggesting that reduced intestinal barrier integrity is the mechanism underlying this pleiotropic effect^45^. In addition, Haller et al. reported significant correlations between the p.Ala391Thr (rs13107325) *SLC39A8* variant, low plasma Mn levels, and adolescent idiopathic scoliosis^46^. They also revealed that an *slc39a8* mutant zebrafish line exhibits a phenotype that includes spinal abnormalities, impaired growth, and decreased motor activity, similar to the symptoms reported in human patients^46^. Moreover, Mn treatment rescued motor activity in these mutant zebrafish, suggesting that patients with scoliosis may benefit from dietary intervention. Wang et al. reported that the p.Ala391Thr variant results in a complete loss of SLC39A8 protein expression, leading to a complete absence of cellular uptake of Mn^2+^ and other bivalent ions. In contrast, other variants, such as p.Ala391Ser, which differs by only a single residue, localize correctly to the plasma membrane and retain normal Mn^2+^ transport function. Several other mutations, including p.Ser335Thr and p.Gly350Arg, also localize to the membrane but show no Mn^2+^ uptake, suggesting transport-incompetent configurations. Additionally, mutations such as p.Thr308Met and p.Gln364Arg selectively impair Mn^2+^ transport while retaining Zn^2+^ uptake, indicating altered substrate specificity. These findings underscore the critical role of SLC39A8 expression and localization in maintaining Mn homeostasis and reveal that even subtle changes in its sequence can result in drastically different functional outcomes^47^.

In contrast to mutations in SLC39A8, mutations in the Mn importer SLC39A14 lead to the accumulation of Mn in the brain and in various other extrahepatic tissues, which is characterized by neurological deficits, motor dysfunction, and gastrointestinal damage^48–51^. Tuschl et al. generated *slc39a14* mutant zebrafish (Slc39a14^U801^) using CRISPR/Cas9 and found that Slc39a14^U801^ larvae have increased levels of systemic Mn and impaired locomotor behavior when exposed to Mn, mimicking the symptoms observed in patients with SLC39A14 deficiency^52^. However, *slc39a14* was expressed in the pronephric duct in zebrafish larvae, suggesting a difference in spatial expression patterns between mammals and zebrafish. Researchers have developed conditional knockout mouse models to better understand the mechanisms by which SLC39A14 mediates Mn homeostasis. For example, we found that liver-specific *Slc39a14* knockout mice (Slc39a14^Alb/Alb^) do not accumulate Mn when fed a diet containing normal concentrations of Mn (10 ppm), suggesting that the loss of Slc39a14 in the liver does not induce spontaneous systemic Mn overload and progressive neuromotor deficits, which are typical phenotypes of global *Slc39a14* knockout mice; in contrast, when these mice are fed a high-Mn diet containing 2400 ppm Mn, they present increased Mn levels in the serum, brain, and pancreas, but not in the liver^49^, indicating that SLC39A14 mediates the transport of Mn from the circulation to the liver. More recently, Aydemir et al. developed an intestine-specific *Slc39a14* knockout mouse (*Slc39a14*^Vil1/Vil1^) and found that compared with global *Slc39a14* knockout mice, *Slc39a14*^Vil1/Vil1^ mice exhibit relatively minor accumulation of Mn in the brain and blood under normal dietary conditions; however, when high amounts of Mn are added to the drinking water, *Slc39a14*^Vil1/Vil1^ mice exhibit significant systemic Mn accumulation and exacerbated brain Mn overload, which are associated with clear early signs of manganism, including motor coordination deficits and dysautonomia^53^. This study demonstrates the indispensable role of intestinal Slc39a14 in Mn detoxification.

SLC39A11 is a novel Mn transporter in vertebrates discovered in 2024 by our group, and we observed that *slc39a11* expression in zebrafish is affected by Mn treatment but not by Zn or Fe treatment^54^. Importantly, we also found that Mn accumulates in *slc39a11* mutant zebrafish, as well as in the serum of both global *Slc39a11* knockout and hepatocyte-specific *Slc39a11* knockout mice, suggesting that this metal transporter regulates systemic Mn levels^54^. Interestingly, elevated systemic Mn levels induce oxidative stress and cellular senescence, which are linked to accelerated aging and a shortened lifespan in *slc39a11* mutant zebrafish. Further studies using genetic models are needed to identify the target tissues in which SLC39A11 affects Mn metabolism.

In summary, multiple Mn transporters likely function synergistically to mediate the uptake and excretion of Mn in various tissues (Fig. 3c). SLC39A8 is expressed in the intestinal epithelium, where it absorbs Mn from the small intestine, whereas hepatic SLC39A8, expressed at the apical canalicular membrane, reclaims Mn from the bile to prevent Mn deficiency. SLC39A11 may play a relatively minor role in the uptake of Mn in the liver, whereas SLC39A14 can mediate the uptake of Mn from the blood into hepatocytes in the liver and at the basolateral membrane of enterocytes in the intestine. Finally, SLC30A10 mediates the efflux of excess Mn from neurons, and Mn that is not utilized by the liver is excreted from hepatocytes into the bile via SLC30A10 located at the apical canalicular membrane. Mn in bile is secreted into the small intestine, where it can either undergo enterohepatic recirculation or be eliminated via the feces by SLC30A10 located at the apical membrane of enterocytes. Additional studies using various conditional knockout animal models are needed to better understand the metabolic processes that maintain systemic Mn homeostasis.

## Cellular manganese dynamics

### Intracellular Mn trafficking

While substantial evidence supports a pivotal role for metal transporters in Mn metabolism, emerging studies suggest that additional proteins, which were identified through in vitro analyses, may also facilitate the intracellular transport of Mn. Together, these proteins constitute an integrated regulatory network that orchestrates the movement, distribution, and utilization of Mn in various cellular compartments and organelles (Fig. 3d).

Studies using HeLa cells to investigate the transport activity of SLC30A10 have shown its localization to the plasma membrane and its role in mediating the rapid efflux of Mn^55^. In the absence of functional SLC30A10 proteins (either by knocking out the gene or introducing a loss-of-function mutation), Mn accumulates in Golgi-derived vesicles, ultimately leading to cytotoxicity^11^. These studies highlight the essential role of vesicular trafficking in SLC30A10-mediated Mn metabolism.

SLC39A8 was initially identified by a transcriptomic screening in monocytes upon exposure to inflammatory and infectious stimuli^56^. Subsequent functional studies using *Xenopus* and mouse oocytes showed that this protein can transport various divalent cations, such as Cd^2+^, Zn^2+^, Mn^2+^, and Fe^2+^ ^27,57–59^. Although lacking ATP-binding domains, SLC39A8 mediates active transport by coupling the movement of metal ions to the HCO₃⁻ gradient, as shown using fetal mouse fibroblasts, and has a higher affinity for Mn^2+^ ions than for Cd^2+^ ions^58^. Cell type-specific functions of SLC39A8 have also been reported. In RBL-2H3 cells (a rat-derived basophilic leukemia cell line), knocking down *Slc39a8* expression significantly reduced Cd/Mn uptake, suggesting that this cell line may serve as a valuable model for studying metal transport^60^. In HeLa cells, mutations in *SLC39A8* cause ER retention, impairing mitochondrial Mn^2+^ delivery and superoxide dismutase 2 (SOD2) activity^61^. Together, these findings establish SLC39A8 as a key regulator of cellular Mn homeostasis.

Initial studies in 2005 using FluoZin-3^AM^ fluorescence and ⁶⁵Zn isotopic labeling showed that when expressed in HEK293T cells, SLC39A14 localizes to the plasma membrane, where it mediates Zn transport^62^. Subsequent studies revealed that this transporter has broader substrate specificity beyond Zn. For example, Pinilla-Tenas et al. reported that SLC39A14 can transport Cd and Mn in canine kidney epithelial cells^63^. Interestingly, the *SLC39A14* mRNA can be alternatively spliced to form two isoforms — namely, Zrt- and Irt-related protein (ZIP) ZIP14A and ZIP14B — and these two isoforms are expressed at different levels in various tissues from C57BL/6 mice^64^. In 2008, Girijashanker et al. reported that SLC39A14 is glycosylated and localizes to the apical surface of cells, and that the ZIP14B isoform transports Mn more efficiently than the ZIP14A isoform when it is expressed in MDCK-polarized epithelial cells^64^. Fujishiro et al. found that the siRNA-mediated knockdown of *SLC39A14* in murine kidney tubular cells significantly reduced Mn uptake, suggesting that SLC39A14, located in the S3 segment of proximal tubules, plays an important role in Mn absorption in the kidney^65^. In Caco-2 cells (a human colorectal adenocarcinoma cell line that is widely used as a model for the intestinal epithelial barrier), the loss of *SLC39A14* significantly reduced Mn secretion (defined as Mn transport from the outside of the base to the top) and strongly increased the absorption of Mn (defined as Mn transport from the top to the outside of the base), suggesting that SLC39A14 is the principal transporter that mediates basolateral Mn uptake in intestinal cells^66^. Moreover, *SLC39A14* knockdown has been shown to significantly reduce Mn levels in multiple cell models, including A549 cells, HepG2 cells, human SH-SY5Y neuroblastoma cells, and brain microvascular endothelial cells (BMVECs)^67–70^; these results further support its role as a functional Mn transporter in vitro.

Although several proteins have been shown to function as Mn transporters in vitro, their physiological roles in vivo require further study^71^. A notable example is the putative cation-transporting ATPase 13A2 encoded by the *ATP13A2* gene, in which mutations have been linked to juvenile-onset atypical Parkinson’s disease and Kufor–Rakeb syndrome (OMIM 606693)^72,73^. Although cellular studies have confirmed that ATP13A2 is involved in lysosomal Mn transport^74,75^, to date, no direct evidence has linked these neurological disorders to altered Mn regulation in patients. In addition, several Ca^2+^ transporters, including transmembrane protein 165 (TMEM165), sarco/endoplasmic reticulum calcium ATPase 2 (SERCA2), and ATPase secretory pathway Ca transporting 1 (ATP2C1), have been shown to transport Mn^76–78^. TMEM165 and ATP2C1 are localized to the Golgi apparatus, where they regulate Mn uptake, supporting the activity of Mn-dependent glycosylation enzymes^5^. Moreover, Mn supplementation has been shown to rescue *TMEM165* deficiency-induced glycosylation defects by restoring Mn homeostasis in the Golgi apparatus^79,80^; interestingly, this rescue depends on the ER enzyme SERCA2 but not ATP2C1^78^. On the other hand, ATP2C1 in the Golgi apparatus may help reduce cellular Mn overload via vesicular trafficking^81^. Although in vitro evidence suggests that these proteins can transport Mn, additional physiological studies in humans and/or animal models are needed to confirm this functional role. Moreover, identifying additional Mn transporters will increase our understanding of Mn metabolism at the cellular and systemic levels.

Mapping the intracellular Mn distribution is critical for elucidating the role of Mn in cell biology. Chemical and elemental imaging studies have indicated that intracellular Mn^2+^ is distributed across multiple compartments, including the mitochondria, nucleus, Golgi apparatus, endoplasmic reticulum, and cytosol, and that its abundance and localization are strongly cell type dependent. In particular, neurons often accumulate more Mn, with Mn signals reported predominantly in the nucleus and cytosol and, surprisingly, little in the mitochondria. In contrast, astrocytes exhibit high levels of cytosolic Mn, which is consistent with their substantial ability to bind to cytosolic Mn (e.g., glutamine synthetase)^11^. Datta et al. further developed a reversible, cell-permeable fluorescent probe (M4) that enables the direct visualization of the spatial distribution and dynamic changes in labile Mn^2+^ levels in living cells. They found that under basal conditions without Mn^2+^ supplementation, endogenous labile Mn^2+^ is confined to sparse punctate foci, consistent with a compartmentalized intracellular pool; upon exogenous Mn^2+^ loading, labile Mn^2+^ becomes more broadly distributed, with increased signals observed throughout the cytosol and perinuclear regions in HeLa cells^82^. In the future, researchers should develop Mn^2+^ sensors that target specific organelles to elucidate the dynamic changes and physiological roles of intracellular Mn in different cellular compartments.

### Ion selectivity of manganese transporters

Given that the proteins SLC30A10, SLC39A8, SLC39A11, and SLC39A14 belong to the SLC30A/ZNT and SLC39A/ZIP families, which are canonically associated with zinc transport, an intriguing question arises concerning the molecular basis for their selective transport of Mn. Therefore, researchers have sought to elucidate the mechanisms underlying this selectivity.

SLC30A10 is a member of the SLC30A family (Fig. 3a) and shares sequence similarity with SLC30A1, suggesting a close evolutionary relationship between these two proteins^83^. However, SLC30A10 and SLC30A1 have key differences in their functions, as the former is primarily a Mn transporter, while the latter is primarily a Zn transporter^84–87^. Studies analyzing their predicted structures have provided clues indicating why these two proteins function differently. For example, the histidine residue at position 43 (His-43) in SLC30A1 is important for the ability of this protein to transport Zn and is consistent with the corresponding His conserved in all other SLC30A members^88^; moreover, changing this residue in SLC30A10 to an asparagine (Asn-43) confers the ability to transport Mn. Indeed, an in vitro study using DT40 cells revealed that Asn-43 in SLC30A10 was required for its Mn-transporting function, whereas replacing Asn with His (N43H) abolished the ability of this protein to export Mn^89^. A subsequent study also supported the hypothesis that the Asn-43 residue in the canonical tetrahedral metal-binding site in SLC30A10 is important for Mn transport and further demonstrated that residue 43 is a key position in the protein that mediates the inward transmembrane calcium gradient for Mn exchange when expressed in HEK293T cells^90^. Moreover, other groups reported that the Asp-248, Glu-25, Asn-127, and Asp-40 residues in SLC30A10 are required for its ability to export Mn when expressed in HeLa cells^91^, whereas the Mn transport activity of SLC30A10 with an Asn-43 mutation was similar to that of wild-type SLC30A10 when expressed in various cell types, including hepatic HepG2 cells, neuronal AF5 cells, HEK293 cells, and embryonic fibroblasts^92^. Given that different results have been obtained in various studies, Shen et al. provided a groundbreaking structural and mechanistic understanding of the Mn efflux transporter SLC30A10 using high-resolution cryo-electron microscopy^93^. They discovered a unique and specific manganese-binding site within the cytosolic cavity of the transporter that is formed by residues Asp40, Asn127, Asp248, and Ser252, and showed that the conserved residue Asp40 is critical, as its mutation abolishes Mn^2+^ binding and transport. However, Asn43 has no significant effect on Mn transport by SLC30A10. These findings reveal the molecular basis of manganese homeostasis and provide a structural blueprint for understanding and potentially treating Mn^2+^-related disorders.

In contrast to the well-studied molecular mechanisms of SLC30A10, how other Mn transporters work is less understood. Both SLC39A8 and SLC39A14 are members of the highly conserved LIV-1 subfamily of SLC39A transporters (Fig. 3b). In addition to containing sequences common to those of other SLC39A transporters, this subfamily contains a highly conserved putative metalloproteinase motif in the fifth transmembrane domain (TM-V), which is highly similar to the active site of metalloproteinases^94,95^. Interestingly, in both SLC39A8 and SLC39A14, the first histidine (H) residue in the signature sequence (HEXPHEXGD) in TM-V is replaced by a glutamate (E), which is believed to confer the ability to bind and transport metal ions other than Zn^64,94,96,97^. Baumann et al. constructed a homology-based 3D model of SLC39A8 using the *Bordetella bronchiseptica* ZIP (BbZIP) structure as a template. Through systematic mutagenesis, they established that the unique glutamate residue (E343), which replaces the histidine conserved in most other SLC39A/ZIP transporters mentioned above, along with residue D410, is essential for Mn²⁺ binding and transport. Mutations at these sites completely abolish Mn²⁺ uptake, underscoring their critical roles in metal selectivity^98^. While SLC39A14 shares sequence homology with SLC39A8, they may share only partially similar mechanisms of Mn transport, as these two proteins usually mediate Mn influx from opposite directions. For example, using the HIBCPP cell line, a polarized monolayer model that mimics the blood–cerebrospinal fluid barrier, Morgan et al. showed that SLC39A14 is enriched at the basolateral membrane, whereas SLC39A8 is localized to the apical membrane, suggesting that these proteins play distinct roles in directional Mn transport^99^. Research on systemic manganese metabolism has also revealed that these genes mediate Mn transport in opposite directions. For example, SLC39A14 is expressed on the basolateral membrane of the choroid plexus and intestinal epithelium and functions primarily in the transport of Mn from the blood into epithelial cells, whereas SLC39A8 is expressed in brain microvessels and on the apical membrane of the intestine, where it mainly mediates the uptake of Mn into tissues and organs^100^. Thus, high-resolution structural studies are needed to determine how these proteins transport Mn via a directionally selective cellular localization mechanism.

SLC39A11 (ZIP11), the sole member of the GufA subfamily of SLC39A/ZIP proteins (Fig. 3b), may have evolved from ancestral eukaryotes because it lacks the histidine-rich loop typically observed in SLC39A/ZIP proteins, and it was previously reported to function as a Zn transporter^101,102^. In filamentous cyanobacterium, Mn treatment increased *slc39a11* expression (measured at the mRNA level) to a much greater extent than Zn treatment, suggesting that the pathway regulating *slc39a11* expression is more sensitive to Mn than Zn^103^. Recently, we reported that both zebrafish and mice expressing a loss-of-function Slc39a11 mutant exhibit systemic Mn accumulation^54^. However, precisely how SLC39A11 mediates Mn transport and its ion affinity both in vitro and in vivo warrants further study.

### Metal cross-talk: homeostatic interplay between Mn and other metals

Fe and Mn share several key physicochemical properties, including their ability to adopt multiple oxidation states and engage in redox activity. In their reduced (divalent) state, both Fe and Mn serve as substrates for divalent metal transporters, enabling the cellular uptake and distribution of these essential metal ions^71^. Several classic Fe transporters also have an affinity for Mn, suggesting overlapping pathways for cellular metal uptake. For instance, the Fe transporters DMT1 and the transferrin–transferrin receptor (Tf–TfR) system, which are essential for the import of Fe, also contribute to Mn uptake. Transferrin-bound Mn^3+^ (Tf-Mn^3+^) can enter cells via TfR-mediated endocytosis, followed by its metalloreductase-mediated reduction to form Mn^2+^ and subsequent cytoplasmic transport via endosomal DMT1^104,105^. Both Fe and Mn accumulate in mitochondria, where transporters, such as DMT1 and mitoferrin-1 (Mfrn1), facilitate the mitochondrial import of these ions^106,107^. However, the role of ferroportin (FPN, the only known Fe exporter) in Mn efflux remains controversial^108,109^.

Under normal conditions, Mn and Fe homeostasis are primarily regulated by distinct transporters, but these shared transporters are activated when these ions become imbalanced. For example, hepatic SLC39A14 typically mediates the uptake of Mn from the plasma but can also import NTBI under iron overload conditions^110^. Similarly, in *Slc30a10* knockout mice, impaired Mn export from enterocytes increases the absorption of dietary Mn via DMT1 and SLC40A1, thus exacerbating systemic Mn overload^111^. Interestingly, Mn and Fe have an antagonistic relationship^112^; while each metal can independently induce oxidative stress and neuronal damage in vitro and in vivo, their combined exposure paradoxically mitigates cytotoxicity by upregulating antioxidant defenses^113,114^. This competitive interaction has important therapeutic implications, as Fe supplementation is used to treat Mn-related metabolic disorders, possibly by reducing oxidative damage^115^.

Although Mn and Zn have distinct biochemical properties, they share several notable similarities with respect to their transport and function^116^. Several Mn transporters, including SLC39A8, SLC39A11, and SLC39A14, also mediate the transport of Zn ions. For example, SLC39A8 co-transports Zn and Mn, and its loss of function concurrently reduces renal levels of both metals, highlighting its intricate role in maintaining metal homeostasis^117^. In addition, both Mn and Zn contribute to cellular detoxification, as MnSOD2 in the mitochondria and Cu/Zn-SOD1 in the cytoplasm catalyze the formation of H₂O₂ from superoxides^118^. Altered regulation of both Mn and Zn homeostasis has been implicated in a variety of pathological conditions, including cardiovascular disease, neurodegenerative disease, autism spectrum disorder, and carcinogenesis^119^; thus, future studies should examine the complex interplay between these metals in both physiological and pathological contexts.

Moreover, Mn overload contributes to various forms of toxicity through several mechanisms. Epidemiological studies have shown that Mn neurotoxicity may be exacerbated by concurrent exposure to arsenic (As), as observed in children residing near coal ash storage sites^120^. In addition, studies using experimental models have shown that combined exposure to Mn and lead (Pb) synergistically exacerbates locomotor deficits and alters the gut microbial composition in zebrafish^121^. At the cellular level, Mn-induced oxidative stress disrupts mitochondrial calcium homeostasis by inhibiting Ca efflux, leading to pathological accumulation in the mitochondria^122,123^; this impaired regulation of mitochondrial calcium homeostasis represents a key mechanism underlying Mn-mediated neurotoxicity and mitochondrial dysfunction.

Interestingly, a recent study revealed a novel form of cross-talk between Mn and copper (Cu) that directly regulates cuproptosis^124^. By employing the highly selective activity-based probe CAP-1 for labile Cu(I), Xie et al. demonstrated that Mn(II) exposure does not simply alter total copper uptake but rather reprograms the intracellular copper redox landscape. It achieves this function by upregulating the mitochondrial reductase FDX1, which promotes the reduction of Cu(II) to the more reactive and toxic Cu(I) and simultaneously depletes the primary antioxidant and metal buffer, reduced glutathione (GSH). This dual action synergistically increases labile Cu(I) pools, thereby driving cuproptosis. In contrast, Zn(II) cotreatment antagonizes copper accumulation and toxicity^124^. This Mn–Cu axis highlights a general principle for metal ion interactions: the phenotypic outcomes are often dictated less by the total metal content than by metal speciation, subcellular redox control, and the capacity of cellular ligands to sequester and detoxify reactive metal pools.

## Manganese-dependent physiological signaling

**Fig. 4:**
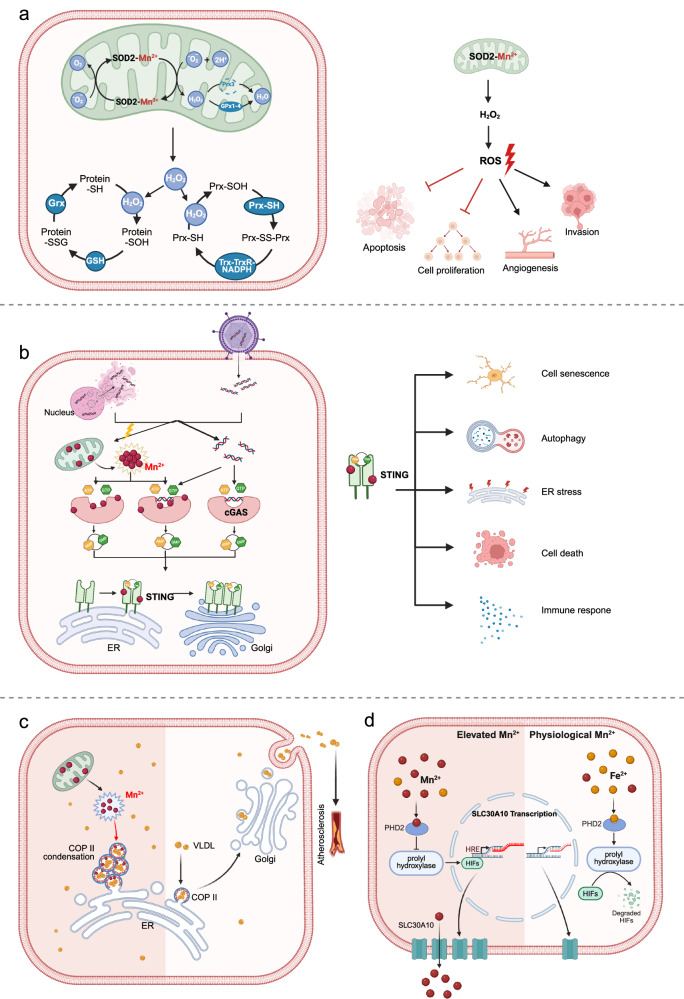
Mn-mediated signaling pathways maintain physiological functions. **a** The Mn-superoxide dismutase (MnSOD) signaling pathway reduces superoxide (O_2_˙^-^) to produce H_2_O_2_, which is then further reduced by either glutathione peroxidase (GPx) 1–4 or peroxiredoxin (Prx) 3 in the mitochondria and by cytosolic Prx 1, 2, 4, and 5 in the cytoplasm to produce water. Oxidized Prx dimers are reduced back to monomers by thioredoxin (Trx) in a process coupled to thioredoxin reductase (TrxR) with NADPH as the electron source. H_2_O_2_ can also react with glutathione (GSH) to form a glutathiolated protein (-SSG) that can be reduced back to the protein thiol by glutathione reductase (Grx). H_2_O_2_ accumulation has been linked to increased angiogenesis and migration and to reduced cell proliferation and apoptosis. **b** Mn is a cofactor in the cGAS–STING pathway. Cellular damage or viral infection releases double-stranded DNA (dsDNA) into the cytosol, which specifically initiates the cGAS–STING pathway with Mn^2+^ as a cofactor. In this process, Mn^2+^ is released from the mitochondria into the cytosol and facilitates the activation of cGAS and STING. STING translocates from the endoplasmic reticulum (ER) to other subcellular compartments (such as the Golgi apparatus, the ER–Golgi intermediate compartment, and lysosomes), where it is activated and initiates multiple cellular processes, including autophagy, cellular senescence, ER stress, cell death, and the immune response. **c** Mn can be mobilized from the mitochondria to activate COPII signaling. Left panel: Mn binds directly to the inner COPII coat and promotes its condensation on the ER surface, and Mn selectively controls hepatic lipoprotein secretion via vesicular trafficking. Right panel: in the absence of Mn, the COPII complex transports lipids into the ER and finally enters the circulation via Golgi trafficking, serving as a risk factor for atherosclerosis. **d** Mn levels can be detected in cells. Left panel: in the presence of elevated intracellular Mn levels, Mn directly inhibits PHD2 by replacing its catalytic Fe and blocking prolyl hydroxylation. Upregulated HIFs bind to the hypoxia response element (HRE) upstream of the *SLC30A10* gene and induce its expression, thereby reducing intracellular Mn levels. Right panel: when intracellular iron (Fe) and Mn levels are balanced, Fe binds to PHD2 (prolyl hydroxylase domain 2) and stabilizes prolyl hydroxylation for the degradation of hypoxia-inducible factors (HIFs). The expression of SLC30A10 is normal.

As an essential trace element, Mn is involved in regulating many key physiological processes. For example, Mn serves as the catalytic site in a wide range of enzymes, including SOD, glutamate synthetase, pyruvate carboxylase, arginase, hydrolase, phosphatases, transferases, dehydrogenases, kinases, peptidases, and decarboxylases; thus, Mn plays a key role in phosphorylation, hydrolysis, carbon metabolism, decarboxylation, and the oxidative stress response^10^. For example, as a coenzyme of SOD2, Mn is involved in redox-sensitive reactions that reduce superoxide (O_2_^•−^) to produce H_2_O_2_, which is then further reduced to produce water by either glutathione peroxidase (GPx) or peroxiredoxins (Prxs)^125,126^. H_2_O_2_ can also react with glutathione (GSH) to form a glutathiolated protein (-SSG) that can be reduced back to the protein thiol by glutathione reductase (Grx)^127^. Increased H_2_O_2_ production following MnSOD2 expression has been linked to reduced apoptosis and cell proliferation, as well as increased angiogenesis and cell migration pathways^128^ (Fig. 4a). From this perspective, cellular Mn levels may serve as a signal for oxidative stress and/or the response to oxidative stress under physiological conditions.

In addition to its role in mediating enzyme activity, Mn also serves as a cofactor in immunological pathways, such as T-cell receptor signaling^129^, immune cell adhesion^130^, and the cGAS/STING pathway, thus playing a key role in the sensitization of tumor immunity and protection against viral infection^131,132^. For example, mitochondria release Mn during cellular damage and microbial infection, which greatly increases the sensitivity of cGAS to double-stranded DNA (dsDNA) and directly activates cGAS to produce the cyclic dinucleotide GMP-AMP (cGAMP)^133^ (Fig. 4b). Mn also facilitates the activation of STING in innate immune activation and antitumor immunity^2,3,134^. In addition, Mn-mediated STING activation plays an important role in the host defense against bacterial infections^135^. For details regarding the effects of Mn homeostasis on the host immune system, the reader is referred to our recent review^12^.

Recently, a novel signaling-based function of Mn distinct from its more commonly recognized roles as a coenzyme and cofactor has been reported. Briefly, Mn directly binds to the inner-coat proteins SEC23/24 in the COPII machinery, thereby selectively regulating the condensation-based transport of lipoproteins and controlling lipoprotein secretion via a distinctive “bell-shaped” function^136^ (Fig. 4c). In addition, the titration of dietary Mn not only tailors the control of circulating lipids in animals but can also significantly revere atherosclerotic plaques, highlighting a potential therapy for treating these potentially deadly cardiovascular lesions^137,138^. In summary, Mn plays a wide range of roles in physiological processes, serving as a coenzyme, a coactivator, and a molecular signal.

In contrast to trace elements with known storage proteins, such as ferritin for iron and metallothionein for zinc, no Mn storage protein has been identified to date, leaving intracellular Mn levels relatively poorly buffered; as a result, Mn sensors play a critical role in detecting Mn concentrations and triggering appropriate homeostatic responses. Studies have shown that elevated levels of intracellular Mn directly inhibit prolyl hydroxylase domain (PHD) enzymes by replacing their catalytic Fe ion and subsequently activate hypoxia-inducible factor (HIF) signaling by blocking the prolyl hydroxylation process, thus increasing the SLC30A10 response^139,140^ (Fig. 4d). The enzyme PHD2 was therefore identified as a critical Mn sensor that controls Mn homeostasis upon Mn overload; however, whether molecules that can sense reduced intracellular Mn levels exist remains unknown. On the other hand, a loss of hepatic Hif2a in *Slc30a10* knockout mice was shown to correct erythropoietin expression and polycythemia, reduce aberrant hepatic gene expression, and reduce excess Mn levels through an unknown mechanism, indicating that hepatic HIF2 plays a key role in the absence of *SLC30A10*^141^. However, a recent study showed that activating HIF1, but not HIF2, regulates the hepatic kynurenine pathway and contributes to neuromotor deficits under both physiological conditions and high-Mn conditions^142^, suggesting that HIF1 and HIF2 control Mn overload-mediated neurotoxicity via distinct mechanisms.

The possible existence of currently unidentified Mn-dependent and/or Mn-mediated signaling pathways represents an exciting avenue for future research. Thus, systematic studies involving a wide range of tissues and organs may reveal novel physiological roles for Mn that are mediated either by direct signaling mechanisms or via indirect involvement in cellular processes. Such discoveries would significantly advance our understanding of the wide array of key biological functions of Mn.

## Regulation of environmental manganese exposure

### Routes of Mn exposure

Although Mn is an essential nutrient, excessive Mn exposure can cause adverse health effects, highlighting the importance of understanding its various sources and associated health risks^143^. Inorganic Mn occurs primarily in the Mn(II), Mn(III), and Mn(IV) oxidation states, with Mn(II) representing the dominant biologically active form because of its high solubility and mobility in environmental and physiological systems^144^. Water-soluble Mn(II) salts, such as MnCl₂ and MnSO₄, are readily absorbed following inhalation or oral exposure, resulting in greater systemic bioavailability than poorly soluble Mn(III) and Mn(IV) oxides (e.g., Mn₂O₃ and MnO₂), which show limited gastrointestinal absorption^33^. Toxicologically, Mn(II) has greater neurotoxic potential than higher oxidation states because of its efficient cellular uptake and disruption of mitochondrial function, dopamine neurotransmission, and redox homeostasis, whereas Mn(III)/Mn(IV) oxides are more strongly associated with pulmonary inflammation and oxidative stress following particulate inhalation^143^.

Occupational settings are the most common source of high exposure to environmental Mn^145^. The first clinical report of Mn toxicity (also known as manganism) was published in the 19th century^1^. Cases of manganism — the majority of which are caused primarily by the inhalation of airborne Mn — have been widely documented globally among miners, dry-cell battery factory workers, smelters, and welders^146,147^. Despite the recognized adverse health consequences, occupational airborne exposure limits for Mn vary among regulatory agencies. The U.S. Occupational Safety and Health Administration (OSHA) has set a permissible exposure limit (PEL) ceiling of 5 mg/m³ for Mn and its compounds in workplace air (https://www.osha.gov/chemical-data). In the European Union, the European Chemicals Agency (ECHA) recommends indicative occupational exposure limit values (IOELVs) of 0.2 mg/m³ (inhalable fraction, 8-h TWA) and 0.05 mg/m³ (respirable fraction) for Mn and inorganic Mn compounds (https://echa.europa.eu). In China, the National Health Commission (NHC) specifies a PC-TWA of 0.15 mg/m³ and a PC-STEL of 0.45 mg/m³ for Mn compounds under the national standard GBZ 2.1-2019 (http://www.nhc.gov.cn). These differences in standards may be at least partially attributable to variations in regional policy frameworks.

Oral ingestion is another common route for toxicity induced by high levels of environmental Mn. Indeed, the ingestion of food or water contaminated with high concentrations of Mn can have adverse health effects. For drinking water, the World Health Organization (WHO) has proposed a health-based guideline value of 0.08 mg/L, while typical background concentrations in surface water and groundwater are usually < 0.03 mg/L. Critically, younger children have relatively high intestinal Mn absorption and reduced biliary Mn expulsion^1,145^. For instance, compared with breastfed infants, infants fed a Mn-containing milk- or soy-based formula have a higher risk of developing this disease^31^. Importantly, high dietary intake of Mn has been linked to attention deficits and learning disabilities in children^148–150^. Moreover, studies in schoolchildren in Bangladesh revealed that Mn levels in drinking water were inversely associated with their academic achievement, particularly in mathematics^151^. Similarly, researchers in Canada and Brazil reported that high Mn concentrations in drinking water were correlated with increased Mn levels in schoolchildren’s hair and were significantly associated with increased hyperactive behaviors and intellectual impairment^151–155^. Furthermore, Italian schoolchildren who lived near a ferroalloy plant were shown to have significantly impaired motor coordination and odor identification following exposure to Mn-contaminated soil^156^.

Organic Mn comprises chemically distinct forms, primarily including organometallic compounds and chelated Mn complexes, which differ from inorganic Mn salts in terms of exposure pathways and toxicokinetics^157^. A representative organomanganese compound is methylcyclopentadienyl Mn tricarbonyl (MMT), which was formerly used as a gasoline antiknock additive, and its exposure is mainly from the inhalation of combustion-derived Mn-containing particles rather than intact MMT itself. Toxicological studies indicate that Mn originating from MMT is metabolically converted into bioavailable inorganic Mn species, enabling their systemic distribution and potential accumulation in the brain, thereby linking its neurotoxicity to Mn^2+^ bioavailability rather than the parent organic structure^33,158^. In contrast, chelated organic Mn compounds used in medical applications, such as mangafodipir, are designed to regulate Mn release following intravenous administration, leading to a controlled tissue distribution and generally lower neurotoxic risk at clinical doses^159^.

**Fig. 5:**
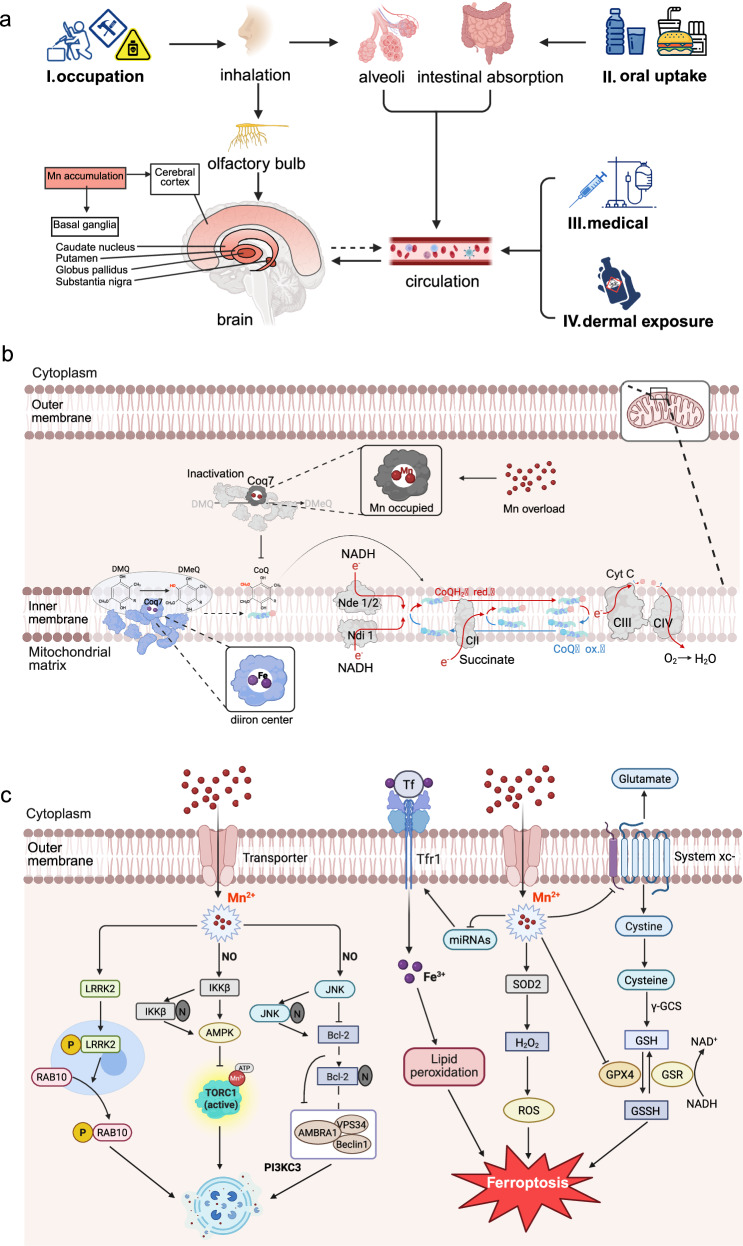
Tissue Mn distribution and toxicity pathways following exposure. **a** Four different routes of Mn exposure (routes I–IV) and systemic Mn distribution are shown. Following occupational exposure (route I), Mn enters primarily via inhalation; inhaled Mn can then directly enter the brain via the olfactory tract, or it can enter the alveoli and then enter the circulation, ultimately reaching the brain by crossing the blood–brain barrier. Mn in the cerebrospinal fluid is released slowly back into the circulation. Upon oral exposure (route II) from drinking Mn-rich water or food, Mn is rapidly absorbed by the intestine and then enters the circulation. The intravenous administration of agents containing high levels of Mn (route III) bypasses the regulatory system in the GI tract, allowing Mn to directly enter the circulation. Finally, Mn exposure through the skin (route IV) is also a risk factor for individuals who come in contact with organic forms of Mn. Excessive amounts of Mn can accumulate in several tissues, including the brain, in which the globus pallidus and substantia nigra in the basal ganglia are the most susceptible brain structures. **b** Mitochondrial toxicity. Overaccumulation of Mn in the mitochondria interferes with the proper metalation of the di-iron hydroxylase Coq7. In turn, loss of Coq7 reduces CoQ (coenzyme Q) production and prevents CoQ-mediated electron transfer in the electron transport chain. The resulting bioenergetic failure drives cell and organismal death. **c** Autophagy dysfunction and ferroptosis through Mn-mediated pathways. Left panel: cellular Mn overload increases the kinase activity of LRRK2, driving the phosphorylation of RAB10 and contributing to impaired autophagy and subsequent neurotoxicity. Cellular Mn overload also induces nitrosative stress, which results in autophagic dysregulation via the Ikkβ–AMPK–mTOR pathway and/or the JNK–Bcl2–Beclin1 pathway. Right panel: cellular Mn overload induces ferroptosis by disrupting iron homeostasis, inducing the accumulation of reactive oxygen species (ROS) and the depletion of glutathione (GSH).

The brain is a primary target organ for Mn toxicity due to its unique pharmacokinetics that govern its absorption, distribution, utilization, and excretion across tissues^160,161^. Upon inhalation, Mn is rapidly absorbed via the olfactory tract and lungs, a process facilitated by high airborne concentrations^160,161^. This absorbed Mn readily reaches the brain via two principal routes: (i) direct transport along the olfactory nerve or (ii) systemic circulation via the alveoli, followed by crossing the blood–brain barrier^162,163^. Furthermore, the exceptionally slow elimination of Mn from the cerebrospinal fluid results in its significant accumulation, thus rendering the brain highly sensitive to systemic Mn overload^164,165^ (Fig. 5a). Notably, the globus pallidus in the subcortical basal ganglia is particularly susceptible to Mn accumulation, with associated neurological disorders comprising the most prevalent symptoms of Mn toxicity^166,167^.

While neurological disorders arising from the accumulation of Mn in the brain represent the hallmark features of manganese toxicity, other organ systems are also adversely affected. For example, the hepatobiliary system primarily mediates Mn clearance, and both hepatic injury and impaired Mn metabolism can accelerate the systemic accumulation of Mn; these changes in turn exacerbate both neurological damage and hepatic pathology, manifesting clinically as a condition called manganese-associated hepatic encephalopathy^168^. In addition to affecting the brain and liver, Mn overload also affects the circulatory system^169^. For example, acute Mn exposure can reduce myocardial contractility and blood pressure, whereas chronic Mn accumulation is associated with polycythemia^85,170^. Notably, recent studies by Huang et al.^171^ and Hu et al.^172^ revealed that excessive Mn exposure is a factor contributing to the risk of sarcopenia^171^, with elevated blood levels of Mn (> 10.6 μg/L) independently associated with sarcopenia in patients receiving hemodialysis^172^.

### Mechanisms underlying Mn toxicity

Several molecular mechanisms underlie Mn-induced neurotoxicity. A primary pathway involves the so-called “mismetallation” of di-iron hydroxylases and the disruption of coenzyme Q biosynthesis during cellular Mn overload, causing mitochondrial bioenergetic failure, which in turn induces premature cell death and ultimately mortality^173^ (Fig. 5b). Emerging evidence suggests that epigenetic changes and impaired regulation of the gut–brain axis may play a role in this process. For example, Wen et al. recently showed that Mn exposure reduces the phosphorylation of the transcription factor SOX2, decreasing the expression of the RNA demethylase FTO, which facilitates the m⁶A-dependent degradation of *GRIN1* and *GRIN3B* transcripts, thus revealing that the SOX2–FTO–GRIN axis is a novel mechanistic target^174^. In addition, Mn exposure activates canonical inflammatory pathways — particularly the NF-κB and ERK1/2 pathways — thereby inducing proinflammatory transcriptional programs in neural and nonneural cells and contributing to the pathogenesis of central nervous system (CNS) injury^175,176^. Furthermore, Mn exposure triggers ER stress, which is characterized by the upregulation of GRP78 and GRP94 expression, with sustained or dysregulated ER stress potentially biasing cells toward apoptosis and cytotoxicity^177^. In astrocytes, Mn-induced ER stress activates the PERK/eIF2α pathway and promotes a detrimental A1-reactive phenotype, linking ER stress to astrocyte-driven inflammation and Mn neurotoxicity^178^.

Another important point is that Mn exposure usually impairs Mn-dependent enzyme activities. For instance, Mn^2+^ physiologically supports the catalytic activity of glutamine synthetase (GS) as an essential divalent metal cofactor, while excessive Mn exposure can toxicologically impair GS expression and/or activity and disrupt the broader glutamate–glutamine (Glu–Gln) cycling network, thereby increasing the glutamate burden and the risk of excitotoxicity^144,179^. Research has shown that astrocytic REST may serve as a potential therapeutic target for Mn-related neurological disorders by alleviating the Mn-induced suppression of EAAT2 and subsequent glutamate excitotoxicity to dopaminergic neurons^180^. Similarly, Mn serves as a structural component of arginase, an enzyme essential for urea cycle function and ammonia detoxification. Altered Mn homeostasis can perturb arginase activity, leading to a metabolic imbalance and directly linking Mn overload to impaired nitrogen metabolism^181^. Complementary studies revealed that transplanting the fecal microbiome from healthy mice into a mouse model of Mn exposure attenuated Mn-induced neurotoxicity, indicating that changes in the microbiome composition and function play a role in inducing a pathogenic cascade^182,183^.

Accumulating evidence from in vitro and in vivo mouse models suggests that ferroptosis plays a role in Mn-induced neurotoxicity^184^. Mn has been shown to trigger ferroptosis via three interconnected pathways (Fig. 5c): (i) the induction of oxidative stress and accumulation of reactive oxygen species (ROS)^185,186^; *ii*) the depletion of GSH and suppression of antioxidant defenses, including downregulation of SLC7A11 and GPX4 expression^187,188^; and (iii) the disruption of Fe homeostasis, leading to iron-dependent lipid peroxidation^189^. Additional mechanisms involve autophagy dysregulation via three processes, namely, *i*) Mn-induced phosphorylation of the LRRK2–RAB10 pathway; *ii*) activation of the S-nitrosylated IKKβ–AMPK–mTOR axis; and *iii*) activation of the JNK–Bcl2–Beclin1 axis^190–192^ (Fig. 5c). Notably, Lai et al. recently performed metabolomic profiling in *Drosophila* and identified biotin metabolism as a key mediator of this process^193^. Biotin depletion exacerbated neurotoxicity in flies lacking the enzyme biotinidase, whereas biotin supplementation reversed Mn-induced pathology in wild-type flies, revealing the therapeutic potential of using biotin to treat Parkinsonism phenotypes. Despite these advances, however, the fundamental mechanisms through which Mn impairs neuronal function remain poorly understood^194^.

Environmental Mn exposure represents a significant public health concern, posing serious threats to our global health and placing a growing burden on our health care system. Thus, primary prevention is paramount, and the most urgent strategy involves the immediate removal of affected individuals from sources of Mn exposure. Consequently, rigorous monitoring of occupational settings, minimizing water and soil contamination, and promoting public health awareness are critical measures needed to reduce the prevalence of Mn-induced toxicity^146,195^.

## Diseases linked to altered Mn regulation

### Inherited disorders

**Fig. 6:**
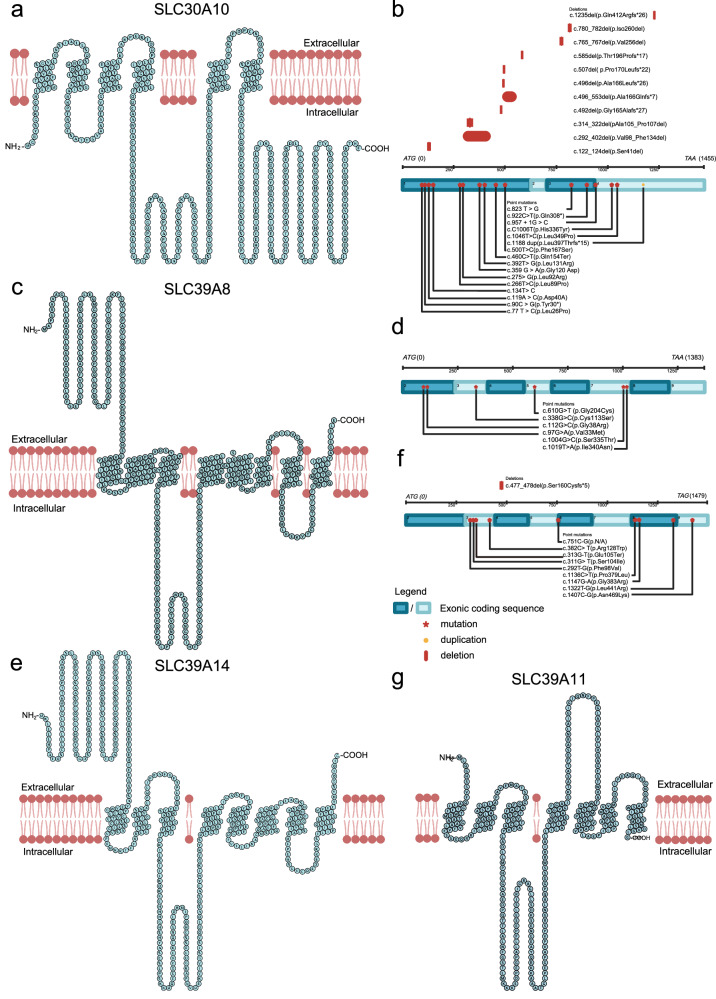
Predicted structures of Mn transporters and mutations identified to date. **a**, **c**, **e**, **g** The topological structures of SLC30A10, SLC39A8, SLC39A14, and SLC39A11 were predicted using www.uniprot.org and were visualized using Protter. **b**, **d**, **f** All mutations in SLC30A10 (B), SLC39A8 (D), and SLC39A14 (F) reported to date in patients. Deletions and point mutations/duplications are listed above and below, respectively, the corresponding mRNAs.

As members of the cation diffusion facilitator (CDF) superfamily, SLC30 proteins typically contain six conserved TM domains^196^ (Fig. 6a). While most SLC30A family members function as zinc transporters, SLC30A10 specifically mediates Mn efflux^197,198^, and its discovery revealed insights into the molecular mechanisms that underlie Mn homeostasis and established the genetic basis for hereditary disorders involving Mn metabolism. In 2008, the first reported case of a rare hereditary disorder involving Mn metabolism was documented in a 12-year-old Arabic girl born to consanguineous parents^199^. She presented with severe hypermanganesemia, hepatic Mn accumulation, Mn deposition in the basal ganglia, and clinical manifestations that included an altered gait, mild hepatic cirrhosis, and polycythemia. Her older brother exhibited identical symptoms before dying at age 18, indicating a lethal autosomal recessive disorder. The genetic basis remained unknown until 2012, when two independent groups identified biallelic *SLC30A10* mutations as the genetic cause of this condition^84,85^. Affected individuals who present with symptoms despite a lack of environmental Mn exposure consistently exhibit severe hypermanganesemia, dystonia–parkinsonism, chronic liver disease, and polycythemia. Prior to this discovery, the molecular mediators of Mn homeostasis were unknown; thus, *SLC30A10* was the first gene linked to hereditary Mn dysregulation, and this discovery fundamentally reshaped the research paradigms concerning metal metabolism^200^. To date, 45 confirmed cases across a wide range of ethnicities have been reported globally (Fig. 6b)^115,201–208^. Current therapies such as chelation therapy and oral iron supplements provide incomplete relief and carry a risk of side effects, underscoring the urgent need for mechanistic studies of SLC30A10-related pathophysiology and the development of molecularly targeted treatments.

SLC39A8 is a key transporter that maintains Mn homeostasis^209^. Like most SLC39A family members, SLC39A8 contains eight TM domains with an extended extracellular N-terminus and a short intracellular C-terminus (Fig. 6c)^210,211^. SLC39A8 is widely expressed in the lungs, kidneys, testes, pancreas, and placenta^56,57,212,213^, and clinical evidence strongly implicates impaired SLC39A8 function in disease development. In 2015, Boycott et al. identified an autosomal recessive disorder caused by a homozygous *SLC39A8* mutation (c.112 G > C, p.Gly38Arg) in 8 patients^209^, with affected individuals exhibiting severe intellectual disability, growth retardation, hypotonia, strabismus, cerebellar atrophy, and critically low blood levels of Mn and Zn. A separate report published in the same year by Park et al. described patients with mutations in the *SLC39A8* gene, including p.Gly38Arg and compound heterozygotes p.Ile340Asn/p.Val33Met/p.Ser335Thr/p.Gly204Cys, who presented with a severe Mn deficiency^214^. Because these patients’ symptoms — skull asymmetry, infantile spasms, dwarfism, brain atrophy, sensory impairments, and glycosylation abnormalities — resembled type II congenital disorder of glycosylation (CDG), this study established a link between Mn deficiency and hereditary CDG, showing that mutations in SLC39A8 can decrease the supply of Mn to the Golgi enzyme β-1-galactosyltransferase, which is essential for glycoprotein biosynthesis. Further expanding on this phenotype, Riley et al. subsequently reported a homozygous *SLC39A8* mutation (c.338 G > C, p.Cys113Ser) causing Leigh-like mitochondrial syndrome, with severe Mn deficiency, growth retardation, dystonia, and seizures^215^. The results of a functional analysis suggested reduced activity of mitochondrial Mn-dependent superoxide dismutase (MnSOD2) and impaired respiratory chain function^215^. Collectively, these clinical cases establish SLC39A8 as an essential Mn transporter and show that mutations can disrupt Mn homeostasis, leading to Mn deficiency and thereby impairing key Mn-dependent processes in glycosylation and mitochondrial antioxidant defense systems.

Genome-wide association studies (GWASs) revealed that loss-of-function mutations in human *SLC39A8* have pleiotropic effects on multiple organ systems and are associated with immune, cardiovascular, neurological, and musculoskeletal disorders^26,216^. To date, clinical data from 14 reported cases worldwide (Fig. 6d) have been used to identify the core phenotype of Mn deficiency, which includes glycosylation defects (resembling type II CDG), mitochondrial dysfunction, intellectual disability, growth retardation, and cerebral atrophy^209,214,215,217^.

Although dietary galactose supplementation has been proposed as a possible therapy^214^, it fails to restore the activity of Mn-dependent metalloenzymes. On the other hand, two patients who received oral Mn supplementation in the form of MnSO_4_ experienced reduced symptoms; within one year of treatment, both patients exhibited significantly improved neurological and motor function, normalized glycosylation rates, normal Mn levels, and a lack of enzyme deficiencies^218^. This report, although it was based on only two patients, helps establish Mn supplementation as a potentially viable treatment for disorders associated with a loss of *SLC39A8*. However, the current lack of established biomarkers for Mn absorption warrants further research to monitor long-term treatment efficacy. From a drug discovery perspective, Damm-Ganamet et al. identified the first small-molecule potentiator of SLC39A8, efavirenz (EFV), through a drug repurposing screen. EFV is proposed to increase the maximal Mn transport capacity of SLC39A8 and to rescue the functional deficits of several disease-associated SLC39A8 variants^219^. These findings validate SLC39A8 as a druggable target and support the use of small-molecule potentiators as a potential therapy for disorders associated with Mn deficiency.

Another SLC39A family member, SLC39A14, is closely related to SLC39A8, as evidenced by common structural features, such as three noncoding exons^211^. The SLC39A14 protein is predicted to contain eight TM domains and an ~54-kDa core, and features a cleavable signal peptide in the N-terminus^96^ (Fig. 6e). Although it was first identified as a transporter of divalent cations (including Zn) and NTBI (which has been implicated in ferroptosis-driven liver fibrosis)^220^, the critical role of SLC39A14 in maintaining Mn homeostasis was established in 2016 when Tuschl et al. reported that homozygous mutations in *SLC39A14* cause systemic Mn accumulation, directly implicating this protein in Mn transport^52^. The broader ion transport features, molecular mechanisms, and physiological functions of SLC39A14 have been reviewed elsewhere^221^; thus, here, we focus specifically on its functional role in transporting Mn.

As mentioned above, individuals carrying homozygous mutations in *SLC39A14* develop systemic Mn accumulation; these patients develop rapidly progressive childhood-onset dystonia–parkinsonism, with clinical findings supported by distinctive brain MRI findings and post-mortem evidence of neurodegeneration^52^. Subsequent reports have confirmed this phenotype; specifically, patients typically present with dystonia, dysarthria, dysphagia, intellectual impairment, and markedly elevated serum Mn levels in early childhood^222–226^ (Fig. 6f). Current management of these patients includes dietary Mn restriction and parenteral chelation therapy with EDTA; however, these interventions have limited efficacy in terms of reversing established neurological deficits^52,224^. Similar to disorders associated with a loss of *SLC30A10*, establishing an effective treatment for patients with *SLC39A14* mutations requires a deeper understanding of the pathways involved and the development of targeted molecular therapies.

SLC39A11 is the fourth identified Mn transporter^54^, and its biological properties remain poorly understood. It is predicted to contain seven TM domains (Fig. 6g), but its primary role is currently unknown. Moreover, to date, no human disorders linked to *SLC39A11* mutations and/or causing altered Mn homeostasis have been reported, highlighting a significant knowledge gap that warrants further investigation.

### Associations with metabolic diseases

**Fig. 7:**
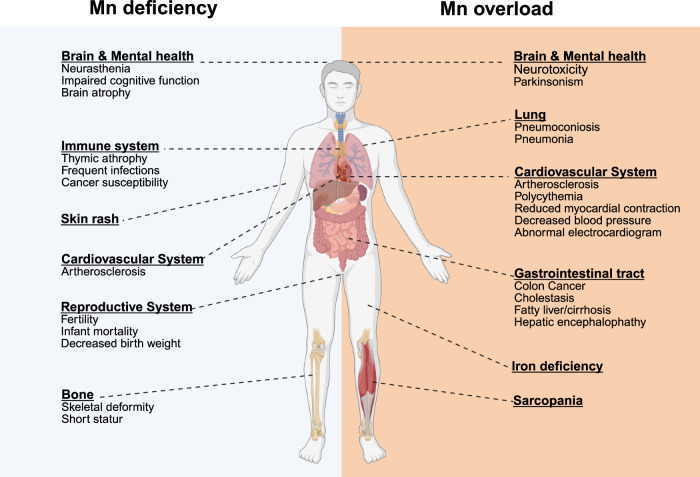
The spectrum of diseases associated with altered Mn homeostasis. Both Mn deficiency (left panel) and Mn overload (right panel) can cause a wide range of diseases and conditions in various tissues and organ systems.

In addition to the neuropsychiatric effects and inherited disorders described above, dysregulated Mn homeostasis has been implicated in a wide range of human pathologies. For example, epidemiological and mechanistic studies performed in recent decades have linked Mn imbalance to glycolipid metabolic disorders, cardiovascular disease, neurological disorders, and immune dysfunction^6,10^ (Fig. 7).

With respect to the underlying mechanism, Mn imbalance promotes oxidative stress by impairing mitochondrial function and promoting excessive ROS production, which can directly exacerbate an underlying metabolic syndrome and its associated pathologies^227,228^. Although a few studies in humans suggest that increased dietary Mn intake can protect against metabolic syndrome^229,230^, this effect appears to be sex specific^231^. Notably, the evidence available to date does not suggest any significant link between circulating or urinary Mn levels and metabolic syndrome^232,233^. Further epidemiological studies are therefore needed to clarify the precise role that the Mn status plays in the development of metabolic syndrome.

Oxidative stress caused by Mn imbalance contributes to impaired pancreatic islet beta cell function, insulin resistance, and ultimately the development of type 2 diabetes mellitus (T2DM) and obesity^234,235^. Animal studies suggest that both Mn-superoxide dismutase (MnSOD) activity and the role of Mn in carbohydrate metabolism are essential for reducing mitochondrial oxidative stress, regulating insulin release and gluconeogenesis, and preventing T2DM^236–238^. However, human epidemiological studies have yielded inconsistent findings, reporting higher^239,240^, lower^241–245^, or unchanged^246^ blood Mn levels in patients with T2DM than in controls. Interestingly, a case-control study in China revealed a “U-shaped” relationship between plasma Mn levels and the T2DM risk, with both reduced and elevated Mn levels associated with an increased risk of T2DM^246^. Thus, although Mn levels appear to affect the development of T2DM, their exact role — either protective or detrimental — remains unclear. Changes in MnSOD activity and oxidative stress have also been implicated in obesity in rodent models. For example, feeding rats a high-fat/high-cholesterol diet significantly reduced MnSOD expression^247^, whereas a loss of MnSOD in mouse adipocytes triggered an adaptive stress response that increased mitochondrial biogenesis and reduced diet-induced obesity^248^. In addition, compared with lean mice, obese mice have been shown to have lower Mn levels in various tissues, such as the liver, small intestine, and bone^249^. Importantly, human epidemiological studies have linked elevated blood Mn concentrations to increased risks of obesity and overweight^233,250^, albeit with inconsistent results reported in different populations^251,252^.

Oxidized low-density lipoprotein (oxLDL) and endothelial dysfunction are key contributors to atherosclerosis, and MnSOD activity has been shown to reduce oxLDL-induced apoptosis in macrophages^253,254^, protect against endothelial dysfunction^255,256^, and inhibit LDL oxidation by endothelial cells^257^. Reduced MnSOD activity is also associated with atherogenesis, and Mn supplementation may help prevent and/or delay disease progression. These findings suggest that Mn levels in the vascular wall matrix may serve as a diagnostic marker for early-stage atherosclerosis^10^. In addition, studies using mouse models have shown that Mn plays a beneficial role in managing hyperlipidemia and atherogenesis^137,138^, highlighting its potential as a novel therapeutic strategy for various forms of cardiovascular disease.

### Associated neurological disorders

Manganism is a neurological disorder associated with elevated blood Mn and Mn deposition in the globus pallidus, most commonly due to environmental exposure. It typically presents as progressive Parkinsonism and motor dysfunction, including bradykinesia, rigidity, dysarthria, dystonia, gait and coordination disturbances, tremor, and, in severe cases, prominent psychotic symptoms^258^. Although manganism shares several clinical similarities with Parkinson’s disease (PD)^259^, key differences exist. For example, Mn exposure typically alters neurotransmitter and dopamine metabolite levels, but without the extensive loss of dopaminergic neurons, which is a characteristic of PD^259^. Unlike PD, which is characterized by α-synuclein aggregation, classical manganism does not involve this pathology, supporting its classification as a distinct disorder^260^. Furthermore, while PD is strongly associated with resting tremors^261^, occupational manganism primarily manifests as impaired cognitive function, psychiatric symptoms, and reduced fine motor control^262^. Therapeutically, manganism is distinguished from PD primarily by its lack of response to levodopa. This clinical hallmark correlates with the relative preservation of the nigrostriatal dopaminergic pathway in patients with manganism. Standard PD medications, including MAO-B inhibitors and anticholinergics, also show limited benefits in the treatment of manganism^260^. The rates of incident PD are higher in counties with high cumulative industrial Mn release than in counties with no/low metal release; similarly, patients with PD residing in high-Mn emission counties have a higher adjusted mortality rate, suggesting that environmental Mn may act as a PD-modifying factor^263^.

Moreover, imbalanced Mn levels may contribute to the pathogenesis of other neurological disorders, such as Huntington’s disease (HD), attention-deficit/hyperactivity disorder (ADHD), and Alzheimer’s disease (AD). For example, clinical and post-mortem evidence suggests altered Mn homeostasis in patients with HD, with reports of elevated Mn levels in the cerebrospinal fluid but reduced Mn levels in the substantia nigra. In HD models, striatal Mn accumulation is diminished (including in HD patient-derived neural progenitors and HD mouse striatal cells) and has been linked to impaired XK/Rab11-dependent Mn handling and disrupted Mn-dependent ATM–p53 signaling, with Mn sensitivity varying by lineage and developmental stage^264^.

A meta-analysis reported significantly higher overall Mn levels (based on combined blood and hair measurements) in children with ADHD^265^, while another logistic regression analysis of Korean children further associated abnormal hair Mn levels with an ADHD diagnosis^266^, and a nested case-control study of adolescents in Pennsylvania and North Carolina showed that higher salivary Mn concentrations are linked to an increased incidence of ADHD^267^, suggesting that Mn disturbances may play a role in disease development. Animal studies have shown that developmental Mn exposure leads to persistent attention and sensorimotor deficits, along with dysregulated catecholaminergic signaling in the prefrontal cortex^268^. Aschner et al. proposed that excess Mn may contribute to ADHD-related pathophysiology by disrupting dopaminergic neurotransmission, altering glutamatergic/GABA metabolism, and inducing oxidative stress and neuroinflammation^269^. However, the detailed underlying mechanisms remain unclear and require further investigation.

Accumulating evidence links chronic Mn exposure to AD-related pathology. In a nested pilot study of Italian ferroalloy workers, occupational Mn exposure was associated with elevated plasma Mn levels and a higher diffuse cerebral amyloid-β (Aβ) burden on PET imaging than in controls. Multi-omics profiling further supported an AD-relevant signature, including shifts in blood-based neurodegeneration markers, exposure-associated autoantibody reactivity to AD- and neuroinflammation-linked targets, and perturbations in pathways implicated in neurodegeneration^270^. In an APP/PSEN1 mouse model of AD, systemic Mn exposure increased brain Mn levels and disrupted glutamatergic homeostasis, whereas data from hippocampal slices further suggested impaired glutamate clearance^271^. In cultured primary cells, Mn suppressed astrocytic glutamate handling and downregulated the expression of related genes^272^. Mou et al. integrated peripheral blood transcriptomic data with a set of “manganese metabolism-related genes” and employed a machine learning approach to screen and validate four potential biomarkers for AD (OPTN, HSP90AA1, NDUFS4, and HSPE1)^273^. These findings suggest that sustained Mn exposure may accompany systemic and central biomarker changes that converge on neurodegenerative processes, providing mechanistic insights into the Mn-associated AD risk.

### Immunological implications

Mn plays a crucial role in nutritional immunity, and altered Mn homeostasis can affect carcinogenesis because of the role of Mn in inflammatory and antioxidant processes^274^. The various biological functions of Mn can affect the incidence, progression, proliferation, and suppression of cancer. For instance, reduced Mn levels have been reported in patients with cervical cancer, and a low Mn status has been correlated with both tumor growth and metastasis in patients with epithelial ovarian cancer^274^. Conversely, the Japan Collaborative Cohort Study for the Evaluation of Cancer Risk revealed that high dietary intake of Mn is associated with a decreased risk of liver cancer in men without pre-existing liver disease^275^. Furthermore, altered expression and acetylation of MnSOD were shown to cause aberrant ROS accumulation, promoting tumorigenesis and therapeutic resistance in various cancers^276,277^.

A growing body of evidence obtained from animal studies shows that Mn also plays a critical role in regulating the immune response. For example, intraperitoneal injections of MnCl_2_ in mice increase the production of type I interferon, significantly increasing natural killer (NK) cell activity^278,279^. Conversely, Mn deficiency accelerated tumor progression and metastasis while reducing the number of tumor-infiltrating CD8^+^ T cells^131^. As an essential micronutrient, Mn increases immune function by facilitating antigen uptake and presentation by antigen-presenting cells and promoting germinal center formation during the adaptive immune response^280^. Importantly, these immunostimulatory properties of Mn have been exploited in vaccine development. First, a colloidal manganese salt (MnJ) has been used as an effective adjuvant, increasing both humoral and cellular immunity while stimulating the production of secretory IgA^280^. Second, nanoparticles incorporating Mn^2+^ have been formulated and shown to significantly improve the efficacy of vaccines against multiple pathogens, including influenza viruses^281^, hepatitis B virus^282^, rabies virus^271^, and novel coronaviruses^283–285^, in different animal models.

Researchers are also currently exploring Mn-based pharmaceuticals, with Mn-based nanomaterials emerging as promising tools for tumor immunotherapy due to their unique immunomodulatory properties^286–288^. For instance, Liang et al. recently developed a near-infrared-II (NIR-II)-responsive plasmonic gold blackbody nanoagonist coated with polydopamine (PDA) to chelate Mn²⁺ (AuPB@PDA/Mn); upon irradiation, this compound generates localized hyperthermia and releases Mn^2+^, significantly increasing immunogenic cell death^289^. Similarly, Wang et al. designed a Mn^2+^-bound bovine serum albumin (BSA)/ferritin-based nanoagonist that enhances the tumor-specific T-cell-mediated immune response against weakly immunogenic solid tumors^290^. Mechanistically, Mn-based nanomaterials have been shown to accumulate in the tumor microenvironment, where they can either directly or indirectly damage tumor cell DNA and/or mitochondria, triggering programmed cell death^291–293^. In addition, these nanomaterials promote the accumulation of cytoplasmic double-stranded DNA (dsDNA), activating the cGAS–STING pathway and increasing the innate immune response. This process includes the production of type I interferon, the secretion of pro-inflammatory cytokines, the maturation of dendritic cells, the infiltration of cytotoxic T lymphocytes, and the recruitment of NK cells^294–296^. Notably, Mn^2+^-mediated activation of the cGAS–STING pathway has been widely reported to amplify the anti-tumor response to a range of immunotherapies^132,297–305^, as comprehensively reviewed recently by Zhang et al.^306^. The growing importance of metal ions in immunomodulation has spurred the emergence of two new fields, metalloimmunology and metalloimmunotherapy, thus highlighting their potential to transform cancer immunotherapy^297,307,308^.

Given the structural simplicity and stability of Mn ions, Mn^2+^-based monotherapies and combination treatment regimens (including immunotherapy) hold significant clinical promise. However, careful monitoring and additional studies are essential during therapeutic application and when initiating large-scale clinical use to minimize the risk of altering Mn homeostasis.

## Conclusions and future perspectives

Excess exposure to environmental Mn (i.e., manganism) and genetic mutations in Mn transporters constitute the primary etiological factors in Mn metabolic disorders. Pediatric populations are particularly vulnerable to both environmental and genetic factors, resulting in diseases that frequently manifest early and often persist throughout life, thus underscoring the urgent need for increased societal awareness and interdisciplinary research efforts.

Although recent advances in the study of Mn biology have revolutionized our understanding of its physiological roles, several critical knowledge gaps remain. First, despite the development of a multivariate gray matter analysis for detecting risks among Mn-exposed welders^309^, reliable biomarkers for Mn accumulation in humans are still needed for early detection and intervention. Second, the complex interplay between Mn and other essential metals, such as Fe, Zn, and Cu, requires further investigation. For example, a growing body of evidence indicates that Mn disrupts Fe homeostasis^310^, for instance, by downregulating the expression of amyloid precursor protein and H-ferritin, leading to impaired iron metabolism and increased neurotoxic oxidative stress^311^. Nevertheless, a key question to be addressed is whether Mn toxicity results primarily from the direct effects of excess Mn or is due to a secondary consequence of altered systemic metal homeostasis.

With respect to genetic disorders affecting Mn metabolism, identifying the molecular mechanisms involving transporter genes should be prioritized, as a comprehensive understanding of the related signaling pathways is essential to determine whether an interconnected network exists among these transporters to cooperatively regulate systemic Mn homeostasis. Importantly, these studies may reveal key regulatory genes that may then serve as therapeutic targets for drug development. Several urgent questions remain. First, although SLC30A10 mediates the release of Mn from the brain and SLC39A8 facilitates its uptake^312^, the precise mechanisms that regulate neuronal Mn transport and accumulation remain poorly understood. Second, in SLC30A10-deficient patients, the severity of liver injury is correlated with hepatic Mn accumulation, yet whether this pathology is linked to the Mn–COPII-mediated retention of lipoproteins in hepatocytes is currently unknown. A third question is related to the pleiotropic effects of *SLC39A8* mutations. Given that the rs13107325 (p.Ala391Thr) variant has been linked to 22 clinical phenotypes^44,313^, an important question is whether this pleiotropy reflects the diverse roles of Mn as an enzymatic cofactor or a common downstream signaling mechanism. Addressing these questions will significantly advance our understanding of Mn-related pathophysiology and may lead to new therapeutic opportunities.

Notably, zebrafish have emerged as a powerful model for studying Mn homeostasis. Wild-type zebrafish embryos serve as a robust system for investigating manganism^314^, while zebrafish carrying mutations in the *slc30a10*, *slc39a8*, and *slc39a14* genes recapitulate many of the clinical phenotypes observed in humans^36,46,52^. Interestingly, *slc39a11* mutants have validated findings obtained from human GWAS analyses^54^. Indeed, as a model system, zebrafish embryos offer several unique advantages, including being optically transparent, allowing the real-time visualization of Mn regulatory proteins using fluorescence in specific cell types and tissues, and representing a promising strategy for future mechanistic studies.

In summary, this review synthesizes our current knowledge regarding Mn signaling pathways under physiological and pathological conditions, encompassing both manganism and inherited Mn transportopathies. The goal of this framework is to provide a comprehensive understanding of Mn metabolism in both health and disease while stimulating further research designed to develop novel preventive and therapeutic strategies.
